# Association of Habitual Alcohol Consumption With Long-term Risk of Type 2 Diabetes Among Women With a History of Gestational Diabetes

**DOI:** 10.1001/jamanetworkopen.2021.24669

**Published:** 2021-09-09

**Authors:** Stefanie N. Hinkle, Wei Bao, Jing Wu, Yangbo Sun, Sylvia H. Ley, Deirdre K. Tobias, Frank Qian, Shristi Rawal, Yeyi Zhu, Jorge E. Chavarro, Frank B. Hu, Cuilin Zhang

**Affiliations:** 1 Epidemiology Branch, Division of Intramural Population Health Research, Eunice Kennedy Shriver National Institute of Child Health and Human Development, National Institutes of Health, Bethesda, Maryland; 2 Department of Epidemiology, College of Public Health, University of Iowa, Iowa City; 3 Glotech Inc, Rockville, Maryland; 4 Department of Preventive Medicine, The University of Tennessee Health Science Center, Memphis; 5 Department of Epidemiology, Tulane University School of Public Health and Tropical Medicine, New Orleans, Louisiana; 6 Division of Preventive Medicine, Department of Medicine, Brigham and Women’s Hospital, Harvard Medical School, Boston, Massachusetts; 7 Department of Nutrition, Harvard TH Chan School of Public Health, Boston, Massachusetts; 8 Department of Medicine, Beth Israel Deaconess Medical Center, Harvard Medical School, Boston, Massachusetts; 9 Department of Clinical and Preventive Nutrition Sciences, School of Health Professions, Rutgers University, Newark, New Jersey; 10 Division of Research, Kaiser Permanente Northern California, Oakland; 11 Department of Epidemiology, Harvard TH Chan School of Public Health, Boston, Massachusetts; 12 Channing Division of Network Medicine, Department of Medicine, Brigham and Women's Hospital, Harvard Medical School, Boston, Massachusetts

## Abstract

**Importance:**

Women with gestational diabetes are at high risk for type 2 diabetes. Identifying modifiable dietary and lifestyle factors, such as alcohol intake, that can be useful in delaying or preventing progression to overt type 2 diabetes is of particular interest.

**Objective:**

To evaluate the association between alcohol consumption and risk for type 2 diabetes among women with a history of gestational diabetes.

**Design, Setting, and Participants:**

This cohort study included women from the Nurses’ Health Study II cohort who reported a history of gestational diabetes and were followed up from January 1, 1991, to December 31, 2017, as part of the Diabetes & Women’s Health Study. Data analysis was performed from 2020 to 2021.

**Exposures:**

Dietary intakes, including alcohol, were assessed every 4 years using validated food-frequency questionnaires.

**Main Outcomes and Measures:**

Multivariable Cox proportional hazards regression models were used to estimate hazard ratios (HRs) and 95% CIs for the association of alcohol intake with risk for incident type 2 diabetes after a pregnancy during which gestational diabetes was diagnosed.

**Results:**

A total of 4740 women were included in the study; the mean (SD) age at baseline was 38.2 (5.0) years, and the median follow-up time was 24 years (interquartile range, 18-28 years), resulting in 78 328 person-years of follow-up. During this period, 897 incident cases of type 2 diabetes were reported. After adjustment for major dietary and lifestyle factors, compared with women who did not consume any alcohol, only alcohol consumption of 5.0 to 14.9 g/d was associated with decreased risk for incident type 2 diabetes (HR, 0.45; 95% CI, 0.33-0.61); there was no association of alcohol consumption of 0.1 to 4.9 g/d or 15.0 g/d or more (maximum, 74.2 g/d) with risk of type 2 diabetes (0.1 to 4.9 g/d: HR, 0.87 [95% CI, 0.73-1.03]; ≥15.0 g/d: HR, 0.62 [95% CI, 0.37-1.04]). After additional adjustment for body mass index, women who reported alcohol consumption of 5.0 to 14.9 g/d had a 41% lower risk for developing incident type 2 diabetes (HR, 0.59; 95% CI, 0.42-0.81); consumption of 0.1 to 4.9 g/d and consumption of 15.0 g/d or more were still not associated with risk of type 2 diabetes, but the results were attenuated (0.1-4.9 g/d: HR, 1.02 [95% CI, 0.85-1.23]; ≥15.0 g/d: HR, 0.75 [95% CI, 0.42-1.33]).

**Conclusions and Relevance:**

In this cohort study, among women with a history of gestational diabetes, usual alcohol intake of 5.0 to 14.9 g/d (approximately 0.5-1 drinks per day) was associated with a lower risk for type 2 diabetes. These findings should be interpreted in the context of other known risks and benefits of alcohol consumption when considering clinical recommendations for individual women with a history of gestational diabetes.

## Key Points

**Question:**

Among women with a history of gestational diabetes, is there an association of habitual alcohol intake with subsequent risk for type 2 diabetes?

**Findings:**

In this cohort study of 4740 women with a history of gestational diabetes, after adjustment for major dietary and lifestyle factors and body mass index, women who consumed 5.0-14.9 g/d had a 41% lower risk for developing incident type 2 diabetes compared with those who did not consume any alcohol.

**Meaning:**

These findings should be interpreted in the context of other known risks and benefits of alcohol consumption when considering clinical recommendations for individual women with a history of gestational diabetes.

## Teaser

This cohort study uses data from the Nurses’ Health Study II to assess the association between habitual alcohol consumption and subsequent risk for type 2 diabetes among women with a history of gestational diabetes.

## Introduction

Prevention of type 2 diabetes is a recognized public health priority.^1^ Women with a history of gestational diabetes have an especially high risk for type 2 diabetes, with a more than 7-fold increased risk compared with women without a history gestational diabetes.^2,3,4^ The role of major risk factors, particularly modifiable dietary and lifestyle factors, in delaying or preventing progression from gestational diabetes to overt type 2 diabetes, is of particular interest. One such factor includes light to moderate alcohol consumption, which has been consistently associated with a lower risk for type 2 diabetes,^5^ particularly in women from the general population.^6^ However, it is unclear whether such findings translate to women with a history of gestational diabetes.

Hendriks^7^ hypothesized that light to moderate alcohol consumption may improve glucose metabolism and insulin sensitivity through modulation of adipokine expression and reduced systemic inflammation. However, because of known risks, heavy alcohol consumption is not recommended.^8,9^ In this study, we used data from a large, prospective cohort with up to 27 years of follow-up to examine associations of habitual alcohol consumption with subsequent risk for type 2 diabetes risk among women with a history of gestational diabetes.

## Methods

### Study Population

The analytical population for this cohort study comprised 4740 women with a history of gestational diabetes in the Nurses’ Health Study II (NHSII) as part of the Diabetes & Women’s Health Study.^10^ The protocol for this study was approved by the Institutional Review Board of the Brigham and Women’s Hospital and Human Subjects Committee Review Board of the Harvard TH Chan School of Public Health in accordance with the Declaration of Helsinki.^11^ According to the informed consent document signed by NHSII participants, return of completed questionnaires implied consent to use the data in ongoing health research. This study followed the Strengthening the Reporting of Observational Studies in Epidemiology (STROBE) reporting guideline for cohort studies.

The NHSII was established in 1989 and is an ongoing prospective cohort study of 116 430 female nurses aged 24 to 44 years at enrollment.^10^ Participants received biennial questionnaires on health-related behaviors and disease outcomes. The follow-up rate as of 2017 for women with gestational diabetes was 88%. Participants in the NHSII were identified as having a history of gestational diabetes based on self-report on the 1991 questionnaire, incident gestational diabetes reported on biennial questionnaires through 2001, or report of a physician’s diagnosis of gestational diabetes on the 2009 pregnancy questionnaire. A high level of surveillance for gestational diabetes has been confirmed in the NHSII, with 83% of women reporting that they had undergone a glucose challenge test during pregnancy and 100% reporting frequent prenatal urine screening.^12^ Participants were excluded from analyses if they reported chronic disease (type 2 diabetes, cardiovascular disease, or cancer [not including nonmelanoma skin cancers]) before the pregnancy during which gestational diabetes was diagnosed or before return of their first post–gestational diabetes food frequency questionnaire (FFQ), had a multiple-gestation pregnancy, or did not return any post–gestational diabetes FFQs.

### Exposure Measurement

Every 4 years since 1991, participants reported the prior year’s dietary intake using a validated semiquantitative FFQ.^13,14,15,16^ For total alcohol intake in kilocalories, the adjusted FFQ intraclass correlation coefficient was 0.91, and the Spearman correlation coefficient compared with 7-day dietary records was 0.86.^16^ Participants were asked how often, on average, over the previous year they consumed beer (regular and light beer), wine (red and white wine), and liquor. Alcohol consumption (in grams per day) was calculated as the sum of daily drinks multiplied by average alcohol content (beer, 12.8 g/12-oz serving; light beer, 11.3 g/12-oz serving; wine, 11.0 g/4-oz serving; and liquor, 14.0 g/serving).^17^ Portion size for wine was increased to 5 oz (148 mL) in 2003, and alcohol content was adjusted accordingly. Women were grouped into 4 alcohol categories: women who reported no alcohol consumption and those reporting alcohol consumption of 0.1 to 4.9 g/d, 5.0 to 14.9 g/d, or 15.0 g/d or more. For analyses on alcohol type, women were grouped according to servings to better reflect the distribution of intake: none, 1 serving per month, 2 to 3 servings per month, 1 to 2 servings per week, and 3 or more servings per week. Because liquor consumption was low, the highest consumption category was 1 or more servings per week.

To represent habitual diet after a pregnancy during which gestational diabetes was diagnosed and to reduce measurement error,^18^ cumulative mean alcohol consumption was calculated based on each post–gestational diabetes FFQ; for example, the 1999 cumulative mean amount was obtained using intake reported in 1991, 1995, and 1999. If a woman was pregnant when she completed the FFQ, the FFQ was considered missing.

### Covariate Assessment

Covariates were chosen a priori based on prior knowledge as factors associated with type 2 diabetes or with lifestyle and therefore potentially also with alcohol intake. Information on age, weight, race/ethnicity, family history of diabetes, smoking status, age at first birth, use of oral contraceptives, and menopausal status was derived from the responses on biennial questionnaires. Covariate status was updated over time during follow-up. Parity was defined as the number of pregnancies lasting more than 6 months. Diet quality was characterized using the Alternate Healthy Eating Index 2010 (AHEI-2010)^19^ with alcohol omitted. Total physical activity in metabolic equivalents was ascertained by reported frequency of engaging in common recreational activities.^20^ Body mass index (BMI) was calculated as weight in kilograms divided by height in meters squared.^21^ Lactation history was requested in 1993, 1997, and 2003; total duration was calculated based on the number of months that women reported complete cessation of breastfeeding after each birth.

### Outcome Measurement

Participants who reported physician-diagnosed type 2 diabetes on a biennial questionnaire were mailed a supplemental questionnaire regarding symptoms, diagnostic tests, and hypoglycemic therapy to confirm self-reported diagnoses. Diagnoses were confirmed if at least 1 of the following was reported^22^: (1) at least 1 symptom of diabetes (excessive thirst, polyuria, weight loss, or hunger) plus a fasting glucose concentration of 7.8 mmol/L or greater (to convert to mg/dL, divide by 0.0555) or random glucose concentration of 11.1 mmol/L or greater; (2) in the absence of symptoms, at least 2 elevated glucose concentrations on different occasions (fasting glucose concentration ≥7.8 mmol/L, random glucose concentration ≥11.1 mmol/L, and/or 2-hour postload glucose concentration ≥11.1 mmol/L on an oral glucose tolerance test); or (3) treatment with insulin or oral hypoglycemic medication. For reports of gestational diabetes after 1998, revised criteria were applied using a fasting glucose concentration cutoff of 7.0 mmol/L.^23^ A subgroup validation study conducted in a similar cohort of female nurses in the US reported a high accuracy rate of 98% when our classification was compared with medical records.^24^

### Statistical Analysis

Data analysis was performed from 2020 to 2021. We defined baseline as the questionnaire period in which participants first reported a pregnancy with gestational diabetes (ie, 1991 for prevalent gestational diabetes and the year of the index pregnancy for incident gestational diabetes). We compared categories of alcohol consumption using analysis of variance for continuous variables and the χ^2^ test for categorical variables.

We computed follow-up time from the date of gestational diabetes diagnosis to a diagnosis of type 2 diabetes, death, or return of last questionnaire, whichever came first. We stopped updating exposure, diet, and physical activity covariate status if a participant reported incident cardiovascular disease or cancer. We used Cox proportional hazards regression models to estimate hazard ratios (HRs) and 95% CIs for the association of risk of type 2 diabetes with total alcohol consumption and consumption of liquor, beer, and wine. Model covariates included age, parity (1, 2, 3, or ≥4), age at first live birth (≤24, 25-29, or ≥30 years), race/ethnicity (White, Black, Hispanic, Asian, or other), family history of diabetes (yes or no), oral contraceptive use (current, former, or never), menopausal status (premenopausal or postmenopausal), cigarette smoking status (current, former, or never), lactation length (<1 month, 1-6 months, >6 to 12 months, >12 to 24 months, or >24 months), physical activity level (quartiles), total energy intake (quartiles), AHEI-2010 diet quality score without the alcohol component (quartiles), coffee consumption (quartiles), and tea consumption (quartiles). Sugar-sweetened beverages were not included in the models because they are a component of the AHEI-2010. We included updated BMI (continuous) in the model separately because directionality between alcohol intake and BMI is not clear. We also included a sensitivity analysis based on BMI at baseline. Associations by alcohol type were adjusted for intake of other types of alcohol (ie, liquor model included beer and wine).

Tests for multiplicative interaction were performed to evaluate modification of associations by factors associated with type 2 diabetes, including family history of type 2 diabetes (yes or no), diet quality (ie, AHEI-2010 score, at or above median or below median), physical activity (at or above median or below median), and obesity (BMI <30 or ≥30); we conducted stratified analyses by these factors. To address potential bias by medical surveillance for type 2 diabetes, we conducted a sensitivity analysis restricting cases of type 2 diabetes to reports of at least 1 symptom of diabetes by participants at the time of diagnosis. We excluded cases of type 2 diabetes reported within the first 2 years of follow-up and women with total alcohol consumption of 30.0 g/d or more because few women (0.7%) consumed amounts of alcohol above this threshold. All statistical analyses were performed using SAS, version 9.4 (SAS Institute Inc). A 2-sided *P* < .05 was considered to be statistically significant.

## Results

A total of 4740 women were included in the study; the mean (SD) age at baseline was 38.2 (5.0) years, and the median follow-up time was 24 years (interquartile range [IQR], 18-28 years). The median time from diagnosis of gestational diabetes to development of type 2 diabetes was 16 years (IQR, 12-22 years). A total of 897 cases of incident type 2 diabetes (18.9%) among the 4740 participants were identified, contributing 78 328 person-years of follow-up. At baseline, 2386 women (50.3%) reported no alcohol consumption in the previous month. Among women who consumed alcohol, median total intake was 2.3 g/d (IQR, 1.1-5.6 g/d), approximately equivalent to 1 drink per week. A total of 113 women (2.4%) reported alcohol consumption of 15.0 g/d or more. The types of alcohol most frequently consumed were beer (median, 0.9 g/d; IQR, 0-1.7 g/d) and wine (median, 0.7 g/d; IQR, 0.4-1.6 g/d). Women who consumed greater amounts of alcohol at baseline were more likely to be older, to be White individuals, to currently be using oral contraceptives, to have current smoking status, to have lower BMI, and to be more physically active; they also consumed more total calories and more coffee but fewer sugar-sweetened beverages and less tea (Table 1). The median change in alcohol consumption among all alcohol consumption groups during follow-up was 0 g/d (0-1.9 g/d).

**Table 1.** Age-Standardized Baseline Participant Characteristics According to Alcohol Consumption Among Women With a History of Gestational Diabetes

Characteristic	Baseline alcohol consumption^a^			
	0 g/d (n = 2386)	0.1-4.9 g/d (n = 1700)	5.0-14.9 g/d (n = 541)	≥15.0 g/d (n = 113)
Age, mean (SD), y				
At baseline	38.1 (5.0)	38.0 (4.9)	38.7 (5.3)	39.6 (4.8)
At first birth	27.2 (5.0)	27.6 (4.9)	28.0 (5.2)	27.8 (4.6)
At index gestational diabetes pregnancy	32.3 (4.9)	32.1 (4.6)	33.1 (5.0)	33.3 (5.0)
Race/ethnicity, No. (%)				
White	2110 (88.4)	1589 (93.4)	504 (93.6)	111 (96.5)
Black	53 (2.2)	18 (1.1)	5 (1.0)	0
Hispanic	50 (2.1)	24 (1.4)	10 (1.8)	1 (2.7)
Asian	101 (4.3)	17 (1.0)	1 (0.2)	0
Other	Unknown	Unknown	Unknown	Unknown
Family history of diabetes, No. (%)	667 (28.0)	442 (26.0)	130 (24.2)	33 (28.5)
Parity, No. (%)				
1	459 (19.7)	323 (19.2)	106 (19.5)	18 (16.4)
2	1042 (43.6)	777 (45.7)	267 (49.5)	50 (53.5)
3	562 (23.6)	422 (24.9)	117 (21.7)	23 (20.1)
≥4	268 (11.3)	152 (9,0)	44 (8.0)	10 (8.6)
Oral contraceptive use, No. (%)				
Current	143 (6.0)	131 (7.7)	50 (9.4)	14 (14.2)
Former	1875 (78.6)	1344 (79.0)	419 (77.5)	96 (83.4)
Never	349 (14.6)	215 (12.9)	71 (13.0)	3 (2.4)
Postmenopausal, No. (%)	142 (6.0)	77 (4.9)	29 (4.2)	4 (2.9)
Smoking status, No. (%)				
Current	219 (9.2)	195 (11.4)	75 (13.8)	26 (21.9)
Former	440 (18.5)	450 (26.6)	179 (33.3)	33 (32.0)
Never	1727 (72.3)	1055 (62.0)	287 (52.8)	54 (46.1)
BMI at baseline, mean (SD)	28.1 (6.8)	26.4 (6.0)	24.8 (5.0)	24.7 (4.8)
Physical activity level, mean (SD), MET h/wk	15.4 (19.9)	18.2 (24.3)	21.7 (24.7)	23.2 (35.7)
Length of lactation, mean (SD), mo	15.4 (15.5)	14.4 (13.5)	14.9 (14.2)	13.8 (12.2)
Total calories consumed, mean (SD), kcal/d	1879 (590)	1918 (552)	2030 (555)	2122 (566)
AHEI-2010 score, mean (SD)	42.4 (10.5)	43.7 (10.0)	45.2 (10.6)	43.1 (9.5)
SSB, mean (SD), servings/d	0.6 (1.0)	0.4 (0.8)	0.4 (0.8)	0.3 (0.7)
Coffee consumption, mean (SD), servings/d	1.0 (1.5)	1.4 (1.6)	1.8 (1.6)	2.2 (1.6)
Tea consumption, mean (SD), servings/d	0.8 (1.2)	0.8 (1.1)	0.7 (1.1)	0.5 (0.9)
Alcohol consumption, median (IQR), servings/d				
Total	0.0 (0.0-0.0)	1.8 (0.9-2.7)	8.3 (6.2-11.0)	21.6 (16.5-29.5)
Liquor	0.0 (0.0-0.0)	0.0 (0.0-0.0)	0.0 (0.0-0.1)	0.1 (0.0-0.4)
Beer	0.0 (0.0-0.0)	0.0 (0.0-0.1)	0.1 (0.0-0.4)	0.9 (0.1-1.4)
Wine	0.0 (0.0-0.0)	0.1 (0.0-0.1)	0.3 (0.1-0.5)	0.4 (0.1-0.9)

Abbreviations: AHEI-2010, Alternate Healthy Eating Index 2010 with alcohol component removed; BMI, body mass index (calculated as weight in kilograms divided by height in meters squared); IQR, interquartile range; MET, metabolic equivalent; SSB, sugar-sweetened beverages.

^a^ Data are from the Nurses’ Health Study II (1989-2017).^10^ All values except age are directly standardized to the age distribution of the study population. Baseline was defined as 1991 for prevalent gestational diabetes and the year of the index pregnancy for incident gestational diabetes. Comparisons across categories of alcohol consumption were statistically significant (*P* < .05) for all variables except family history of diabetes.

In the adjusted model including major dietary and lifestyle factors, compared with women who did not consume any alcohol, only alcohol consumption of 5.0 to 14.9 g/d was associated with a decreased risk for incident type 2 diabetes (HR, 0.45; 95% CI, 0.33-0.61); there was no association of alcohol consumption of 0.1 to 4.9 g/d or 15.0 g/d or more (maximum, 74.2 g/d) with risk of type 2 diabetes (0.1 to 4.9 g/d: HR, 0.87 [95% CI, 0.73-1.03]; ≥15.0 g/d: HR, 0.62 [95% CI, 0.37-1.04 ]) (Table 2). After additional adjustment for BMI, women who reported alcohol consumption of 5.0 to 14.9 g/d had a 41% lower risk for developing incident type 2 diabetes (HR, 0.59; 95% CI, 0.42-0.81); consumption of 0.1 to 4.9 g/d and consumption of 15.0 g/d or more were still not associated with risk of type 2 diabetes, but the results were attenuated (0.1-4.9 g/d: HR, 1.02 [95% CI, 0.85-1.23]; ≥15.0 g/d: HR, 0.75 [95% CI, 0.42-1.33]). In analyses stratified by factors associated with type 2 diabetes, HRs did not significantly differ among strata (Table 3). Although estimates differed by obesity status, they were too imprecise for meaningful conclusions to be made.

**Table 2.** Habitual Alcohol Consumption and Risk of Type 2 Diabetes Among Women With a History of Gestational Diabetes

Variable	Cumulative mean consumption of alcohol			
	0 g/d (n = 1558)	0.1-4.9 g/d (n = 2249)	5.0-14.9 g/d (n = 752)	≥15.0 g/d (n = 181)
Total alcohol consumption, median (IQR), g/d	0	1.6 (0.9-2.7)	8.1 (6.4-10.7)	21.3 (17.2-27.3)^a^
Women with type 2 diabetes, No.	386	420	72	19
Person-years	28 712.0	35 642.2	11 400.3	2573.3
Age-adjusted analysis, HR (95% CI)	1 [Reference]	0.79 (0.68-0.92)	0.42 (0.32-0.55)	0.52 (0.32-0.84)
Multivariable analysis, HR (95% CI)^b^	1 [Reference]	0.87 (0.73-1.03)	0.45 (0.33-0.61)	0.62 (0.37-1.04)
Multivariable analysis including BMI, HR (95% CI)^c^	1 [Reference]	1.02 (0.85-1.23)	0.59 (0.42-0.81)	0.75 (0.42-1.33)

Abbreviations: BMI, body mass index; HR, hazard ratio; IQR, interquartile range.

^a^ The upper bound of total alcohol consumption was 74.2 g/d.

^b^ Adjusted for age, parity (1, 2, 3, or ≥4), age at first birth (12-24, 25-29, or ≥30 years), race/ethnicity (White, Black, Hispanic, Asian, or other), family history of diabetes (yes or no), oral contraceptive use (current, former, or never), menopausal status (premenopausal or postmenopausal), cigarette smoking (current, former, or never), lactation length (<1 month, 1-6 months, >6 to 12 months, >12 to 24 months, or >24 months), physical activity level (quartiles), total energy intake (quartiles), Alternate Healthy Eating Index 2010 diet quality score with alcohol component removed (quartiles), coffee consumption (quartiles), tea consumption (quartiles).

^c^ Multivariable model additionally adjusted for BMI (continuous).

**Table 3.** Habitual Alcohol Consumption and Risk of Type 2 Diabetes Among Women With a History of Gestational Diabetes Stratified by Family History of Diabetes, Diet Quality, Physical Activity, and Body Mass Index

Variable	Cumulative mean consumption of alcohol			
	0 g/d	0.1-4.9 g/d	5.0-14.9 g/d	≥15.0 g/d
**Family history of diabetes** ^**a**^				
No				
Women, No.	840	1269	471	125
Women with type 2 diabetes, No.	184	202	32	7
Person-years	16 266	21 385	7091	1726
Multivariable analysis including BMI, HR (95% CI)^b^	1 [Reference]	1.01 (0.77-1.32)	0.55 (0.34-0.89)	0.42 (0.17-1.01)
Yes				
Women, No.	483	776	222	51
Women with type 2 diabetes, No.	142	172	27	9
Person-years	8055	10 347	3104	647
Multivariable analysis including BMI, HR (95% CI)^b^	1 [Reference]	0.94 (0.67-1.32)	0.50 (0.28-0.89)	0.91 (0.35-2.34)
**AHEI-2010 score** ^**a**^				
Less than median score^c^				
Women, No.	639	805	179	43
Women with type 2 diabetes, No.	194	189	17	8
Person-years	13 885	15 555	3871	892
Multivariable analysis including BMI, HR (95% CI)^b^	1 [Reference]	0.93 (0.70-1.23)	0.40 (0.22-0.75)	0.82 (0.32-2.07)
Median score or greater^c^				
Women, No.	684	1240	514	133
Women with type 2 diabetes, No.	132	185	42	8
Person-years	10 437	16 177	6323	1480
Multivariable analysis including BMI, HR (95% CI)^b^	1 [Reference]	1.07 (0.79-1.47)	0.63 (0.39-1.02)	0.65 (0.25-1.69)
**Physical activity** ^**a**^				
Less than median level^d^				
Women, No.	747	930	223	60
Women with type 2 diabetes, No.	207	200	27	8
Person-years	14 047	15 294	3744	928
Multivariable analysis including BMI, HR (95% CI)^b^	1 [Reference]	0.86 (0.65-1.13)	0.53 (0.32-0.88)	0.75 (0.30-1.89)
Median level or greater^d^				
Women, No.	576	1115	470	116
Women with type 2 diabetes, No.	119	174	32	8
Person-years	10 274	16 438	6451	1444
Multivariable analysis including BMI, HR (95% CI)^b^	1 [Reference]	0.98 (0.71-1.36)	0.60 (0.35-1.01)	1.08 (0.46-2.52)
**BMI** ^**a**^				
<30.0				
Women, No.	645	1179	510	131
Women with type 2 diabetes, No.	88	113	23	9
Person-years	17 735	25 769	9157	2134
Multivariable analysis including BMI, HR (95% CI)^b^	1 [Reference]	0.93 (0.64-1.36)	0.53 (0.29-0.98)	1.37 (0.56-3.36)
≥30.0				
Women, No.	678	866	183	45
Women with type 2 diabetes, No.	238	261	36	7
Person-years	6587	5963	1037	238
Multivariable analysis including BMI, HR (95% CI)^b^	1 [Reference]	0.99 (0.77-1.27)	0.61 (0.39-0.96)	0.36 (0.13-1.00)

Abbreviations: AHEI-2010, Alternate Healthy Eating Index 2010; BMI, body mass index (calculated as weight in kilograms divided by height in meters squared); HR, hazard ratio.

^a^ All tests for interaction between alcohol and family history of diabetes, diet quality, physical activity, and BMI were not significant (*P* > .05).

^b^ Adjusted for all variables given in the Statistical Analysis subsection of the Methods section except the variable of interest.

^c^ The median AHEI-2010 score was 44.6 (IQR, 38.4-51.7).

^d^ The median physical activity level was 11.9 metabolic equivalent h/wk (IQR, 5.5-22.7).

Sensitivity analyses for main results yielded consistent findings in general. When adjustments were made for baseline BMI, results were similar. Compared with no alcohol consumption, consumption of 0.1 to 4.9 g/d or 15.0 g/d or more was not associated with risk of type 2 diabetes (0.1-4.9 g/d: HR, 1.02 [95% CI, 0.85-1.23]; ≥15.0 g/d: HR, 0.75 [95% CI, 0.42-1.33]), but consumption of 5.0 to 14.9 g/d was associated with decreased risk (HR, 0.59; 95% CI, 0.42-0.81). Restricting type 2 diabetes cases to reports of at least 1 symptom of diabetes by participants (n = 375) at the time of diagnosis yielded similar results but a slightly stronger association with alcohol consumption of 5.0 to 14.9 g/d (HR, 0.30; 95% CI, 0.16-0.56); for alcohol consumption of 0.1 to 4.9 g/d, the HR was 0.96 (95% CI, 0.73-1.27), indicating no association, and for consumption of 15.0 g/d or more, the HR was 0.49 (95% CI, 0.17-1.40), indicating no association. When limited to women reporting alcohol consumption of less than 30.0 g/d (n = 4727) and adjusted for major dietary and lifestyle factors, alcohol consumption of 15.0 to 29.9 g/d was associated with a 47% lower risk for type 2 diabetes (HR, 0.53; 95% CI, 0.29-0.97); however, there was no association after additional adjustment for BMI (HR, 0.66; 95% CI, 0.34-1.26). Findings were robust to the exclusion of type 2 diabetes diagnoses made during the first 2 years of follow-up. Compared with no alcohol consumption, the fully adjusted HR for consumption of 0.1 to 4.9 g/d was 1.03 (95% CI, 0.85-1.24), indicating no association; for consumption of 5.0 to 14.9 g/d, the HR was 0.59 (95% CI, 0.43-0.82), indicating an association with decreased risk of type 2 diabetes; and for consumption of 15.0 g/d or more, the HR was 0.75 (95% CI, 0.42-1.33), indicating no association.

Analyses by type of alcohol are shown in Table 4. In fully adjusted models, compared with no liquor consumption, consumption of liquor was not associated with risk of type 2 diabetes. Compared with no beer consumption, beer consumption of 1 to 2 servings per week and 3 or more servings per week was associated with decreased risk of type 2 diabetes. Compared with no wine consumption, consumption of 3 or more servings per week was inversely associated with risk of type 2 diabetes in the age-adjusted and multivariable analyses (age-adjusted analysis: HR, 0.46; 95% CI, 0.31-0.67; multivariable analysis: HR, 0.56; 95% CI, 0.37-0.86) but not in a multivariable analysis that included BMI (HR, 0.73; 95% CI, 0.46-1.15).

**Table 4.** Type of Alcohol Consumption and Risk of Type 2 Diabetes Among Women With a History of Gestational Diabetes

Variable	Cumulative mean consumption of alcoholic drinks				
	0	Servings/mo		Servings/wk	
		1	2-3^a^	≥1 or 1-2^b^	≥3
**Liquor**					
Servings, median (IQR)	0	0.7 (0.5-1.0)	2.0 (1.6-2.1)	1.7 (1.2-3.0)	NA
Women, No.	3106	706	645	283	NA
Women with type 2 diabetes, No.	645	118	92	42	NA
Person-years	56 474	8311	9140	4402	NA
Age-adjusted analysis, HR (95% CI)	1 [Reference]	1.26 (1.00-1.58)	1.06 (0.83-1.37)	1.29 (0.90-1.84)	NA
Multivariable analysis, HR (95% CI)^c^	1 [Reference]	1.10 (0.87-1.40)	1.00 (0.76-1.31)	1.20 (0.82-1.77)	NA
Multivariable analysis including BMI, HR (95% CI)^d^	1 [Reference]	1.13 (0.87-1.46)	1.11 (0.83-1.47)	1.10 (0.72-1.68)	NA
**Beer**					
Servings, median (IQR)	0	0.7 (0.5-1.0)	2.0 (2.0-3.1)	1.5 (1.2-2.0)	4.2 (3.3-6.0)
Women, No.	2872	457	810	395	206
Women with type 2 diabetes, No.	626	77	136	37	21
Person-years	49 161	5506	13 268	6255	4138
Age-adjusted analysis, HR (95% CI)	1 [Reference]	1.02 (0.78-1.33)	0.91 (0.73-1.13)	0.53 (0.37-0.77)	0.53 (0.33-0.84)
Multivariable analysis, HR (95% CI)^c^	1 [Reference]	1.11 (0.84-1.47)	0.87 (0.69-1.09)	0.49 (0.34-0.72)	0.55 (0.34-0.89)
Multivariable analysis including BMI, HR (95% CI)^d^	1 [Reference]	0.96 (0.71-1.31)	0.81 (0.63-1.04)	0.55 (0.36-0.83)	0.60 (0.36-1.00)
**Wine**					
Servings, median (IQR)	0	0.7 (0.5-1.0)	2.0 (2.0-3.1)	1.6 (1.2-2.0)	4.4 (3.5-6.0)
Women, No.	1922	572	1163	675	408
Women with type 2 diabetes, No.	452	103	234	73	35
Person-years	36 289	7023	19 270	9429	16 317
Age-adjusted analysis, HR (95% CI)	1 [Reference]	0.98 (0.77-1.25)	0.86 (0.71-1.04)	0.58 (0.44-0.78)	0.46 (0.31-0.67)
Multivariable analysis, HR (95% CI)^c^	1 [Reference]	1.06 (0.82-1.37)	0.95 (0.78-1.17)	0.77 (0.56-1.05)	0.56 (0.37-0.86)
Multivariable analysis including BMI, HR (95% CI)^d^	1 [Reference]	1.16 (0.87-1.54)	1.11 (0.89-1.39)	0.97 (0.69-1.36)	0.73 (0.46-1.15)

Abbreviations: BMI, body mass index; HR, hazard ratio; IQR, interquartile range.

^a^ The 2 to 3 servings per month category includes all values greater than 1 serving per month to less than 1 serving per week and therefore includes some values greater than 1 but less than 2 servings per month. However, for simplicity the label is 2 to 3 servings per month.

^b^ For liquor, column shows data for 1 or more servings; for beer and wine, 1 to 2 servings. The upper bounds for liquor, beer, and wine were 4.9 servings per day, 4.6 servings per day, and 3.2 servings per day, respectively.

^c^ Adjusted for age, parity (1, 2, 3, or ≥4), age at first birth (12-24, 25-29, or ≥30 years), race/ethnicity (White, Black, Hispanic, Asian, or other), family history of diabetes (yes or no), oral contraceptive use (current, former, or never), menopausal status (premenopausal or postmenopausal), cigarette smoking status (current, former, or never), lactation (<1 month, 1-6 months, 6 to 12 months, 12 to 24 months, or >24 months), physical activity (quartiles), total energy intake (quartiles), Alternate Healthy Eating Index 2010 diet quality score (quartiles), coffee consumption (quartiles), tea consumption (quartiles). Each alcohol type model was concurrently adjusted for intake of other types of alcohol (ie, liquor model was adjusted for beer and wine).

^d^ Multivariable model additionally adjusted for BMI (continuous).

## Discussion

In this prospective cohort study of women with a history of gestational diabetes followed up for up to 27 years, alcohol consumption between 5.0 and 14.9 g/d was associated with a 55% lower risk for type 2 diabetes (HR, 0.45; 95% CI, 0.33-0.61) compared with no alcohol consumption. The association was independent of demographic and other major dietary and lifestyle factors. Additional adjustment for BMI slightly attenuated the findings, although alcohol consumption of 5.0 to 14.9 g/d remained associated with a 41% lower risk for type 2 diabetes (HR, 0.59; 95% CI, 0.42-0.81).

Prior studies^6,25^ of alcohol consumption and type 2 diabetes have been conducted mainly among the general population, whereas our study focused on a population of women with a history of gestational diabetes, 18.9% of whom had received a diagnosis of type 2 diabetes during follow-up. In our study, after adjustment for BMI, alcohol consumption of 0.1 to 4.9 g/d was not associated with risk for type 2 diabetes, although consumption of 5.0 to 14.9 g/d was associated with a 41% lower adjusted risk for type 2 diabetes. The latter finding is consistent with a 43% estimated lower risk associated with consumption of 12 to 24 g/d among women in the general population^6^ and a 33% estimated reduction in risk associated with consumption of 5.0 of 14.9 g/d in the entire NHSII cohort.^25^ However, our results for alcohol consumption of 0.1 to 4.9 g/d varied from the overall findings for the NHSII cohort,^25^ in which alcohol consumption of 0.1 to 4.9 g/d was associated with a 20% lower risk for type 2 diabetes; no benefit was observed in our study. Although the reason for such discrepancy is uncertain, there are a few potential contributing factors. Women in the present study were followed up for a longer period; they also had a greater risk for type 2 diabetes, and women with gestational diabetes may have more insulin resistance and more systemic inflammation, potentially requiring a higher threshold for alcohol consumption to demonstrate a benefit.^26^ Also, in the NHSII cohort,^25^ alcohol consumption of 15.0 to 29.9 g/d was associated with a 58% lower risk for type 2 diabetes, which differs from our findings. Only 2.4% of women in our study reported alcohol consumption of 15 g/d or more at baseline, limiting our power to examine risks associated with heavy consumption.

A meta-analysis^27^ of intervention studies revealed that alcohol consumption of 11 to 40 g/d by women was associated with improved insulin sensitivity and hemoglobin A_1c_ levels during a follow-up period of 2 to 12 weeks. Schrieks et al^27^ hypothesized that the effect of alcohol on both limiting glucose concentration and increasing insulin concentration after a meal may be associated with improved glucose control and a lower risk for type 2 diabetes. Also, alcohol consumption of up to 15 g/d in women is associated with increased concentrations of high-density lipoprotein cholesterol and adiponectin,^28^ which in turn may be associated with a lower risk for type 2 diabetes; the causal relationship between these factors is not known.^29,30,31^ In addition, greater alcohol consumption is associated with lower plasma fetuin-A levels, a liver-derived protein that can cause insulin resistance.^32^ Nonetheless, excess alcohol consumption leads to hepatic de novo lipogenesis and promotes the development of fatty liver disease, which may be associated with an increased risk for type 2 diabetes.^33^

In our analyses, habitual alcohol consumption of 5.0 to 14.9 g/d was associated with a lower type 2 diabetes risk independent of BMI, and there was no association between alcohol consumption of 0.1 to 4.9 g/d and 15.0 g/d or more and risk for type 2 diabetes after adjustment for BMI, although the estimates were attenuated. At baseline, women who consumed alcohol had a lower BMI than women who did not consume alcohol; however, a strength of this study was the longitudinal follow-up with updated BMI. Findings regarding the association between habitual alcohol intake and weight gain have varied, with some studies showing no association between light to moderate alcohol consumption and weight gain^34^ and others showing a positive association.^35^

As reported by Huang et al,^36^ previous observational studies have suggested that wine is more strongly associated with a lower risk for type 2 diabetes than beer or liquor. In our analysis, only beer consumption of 1 or more servings per week was associated with lower risk for type 2 diabetes, whereas wine and liquor consumption were not associated with this risk. Results for liquor were imprecise owing to limited consumers. Although some null findings by alcohol type could be attributable to a lack of power, there may also have been differences in behavioral factors by the type of alcohol consumed. Our study was conducted with data from a well-characterized prospective cohort with comprehensive adjustment for lifestyle factors, although we cannot rule out residual confounding. Also, it is possible that there may be more reporting error for wine than for beer because of differences in servings and consumption patterns.

Although our findings suggest that alcohol consumption of 5.0 to 14.9 g/d compared with no consumption is associated with a 41% lower risk for type 2 diabetes among women with a history of gestational diabetes, they should not be interpreted in isolation from other health outcomes. Among those who consume alcohol, consumption of 0.1 to 14.9 g/d has been associated with improved cardiovascular outcomes and lower all-cause mortality,^9^ although consumption levels as low as 5 to 9 g/d have been associated with a modest increase in the risk for breast cancer.^37^ Thus, careful balancing of risks and benefits is needed. Consistent with the 2020 Dietary Guidelines for Americans, which recommend that adults who do not consume alcohol do not initiate drinking,^38^ it may not be prudent for those with a history of gestational diabetes who do not consume alcohol to initiate drinking alcohol solely to reduce their risk for type 2 diabetes.

### Strengths and Limitations

This study has strengths, including the prospective cohort study design, which strengthens the temporal direction of associations; long-term follow-up; a large sample size; a high rate of follow-up; and detailed and repeated assessments of alcohol consumption with validated questionnaires. Our study also included adjustments for multiple potential confounding lifestyle factors, including dietary quality,^39^ physical activity,^40^ lactation history,^41^ and BMI.^42^

This study also has limitations. Although NHSII participants were registered nurses at enrollment, potentially reducing confounding by educational attainment or differential access to health care, residual confounding remains possible. Misclassification of alcohol consumption was possible owing to underreporting; however, alcohol consumption measured by the FFQ has been validated against food diaries and using high-density lipoprotein cholesterol level.^14^ Also, screening bias may exist because women who were more health conscious and therefore visited a physician more regularly may have had a greater chance of receiving a medical diagnosis than those who were less health conscious. However, we found similar results in our sensitivity analyses restricting cases to only those in women experiencing symptoms of type 2 diabetes, which minimizes concerns for this bias. This study consisted predominantly of White women in the US whose alcohol consumption was relatively low, potentially limiting generalizability. Also, we lacked information on binge drinking and whether alcohol was consumed with meals, which may be important if the mechanism is related to postprandial glucose and insulin metabolism; further research is warranted. The sample size may have precluded us from detecting modification of associations by risk factors. Also, statin use may be a confounder; however, because of the small number of women treated with statins in 1999 (the first year statin treatment was assessed), we could not adjust for statin use.

## Conclusions

In this cohort study, an inverse association was observed between alcohol consumption of 5.0 to 14.9 g/d and risk for type 2 diabetes among women with a history of gestational diabetes. Findings should be interpreted in the context of other known risks and benefits of alcohol consumption when considering clinical recommendations for individual women with a history of gestational diabetes.
